# Improving the Management of Hereditary Angioedema

**DOI:** 10.6061/clinics/2018/e354

**Published:** 2018-05-08

**Authors:** Pedro Giavina-Bianchi, Jorge Kalil

**Affiliations:** Divisao de Imunologia Clinica e Alergia, Faculdade de Medicina (FMUSP), Universidade de Sao Paulo, Sao Paulo, SP, BR

Hereditary angioedema (HAE) is a syndrome characterized by recurrent angioedema of the skin and mucosa induced by genetic mutations that result in increased bradykinin concentration in the tissues. HAE presents high morbidity and mortality, especially if not adequately treated, since patients live with the risk of having laryngeal angioedema that can lead to death due to asphyxia. Abdominal attacks are also very disabling, and patients often inadvertently undergo exploratory laparotomies.

There are approximately 10,000 rare diseases, many of which are life threatening and neglected, having problematic diagnosis and poor therapy. HAE is a good example of how this reality can be changed, with the support and partnership of academia, the pharmaceutical industry, government and NGOs, so that barriers are overcome and common goals are achieved. In the last decade, knowledge regarding HAE has increased: approximately 30 consensuses/guidelines were developed and published worldwide, referral centers specialized in the disease were structured, and educational programmes for patients and their relatives were implemented. New drugs were developed, the effectiveness of which have been proven in the prophylaxis and treatment of HAE attacks. As a consequence, better diagnosis and treatment of HAE have improved patients' quality of life and decreased disease morbidity and mortality.

The statement "Medicine is a science and an art" is attributed to Sir William Osler, a physician with numerous scientific contributions, including the recognition of HAE as a hereditary disease. The development of guidelines based on scientific evidence is practicing medicine as a science. Using the guidelines as an orientation, but attending patients with their various phenotypes in a personalized way, is an art. The publication of the "Brazilian Guidelines for the Diagnosis and Treatment of Hereditary Angioedema" in 2011 1,2 was a milestone that boosted the care, education and research in HAE in the country. Knowledge about the disease improved, patient diagnoses increased and were made earlier, and new treatments became available, providing great improvement in HAE management. However, we need to expand the projects that are under development, making them more homogenous and covering all regions of Brazil, as well as promote new initiatives to keep ameliorating the status quo.

In this scenario, a group of experts from the Brazilian Association of Allergy and Immunology (ASBAI) and the Brazilian Group for the Study of Hereditary Angioedema (GEBRAEH) updated the 2011 Guidelines. While 9 specialists from 7 services participated in the first guidelines, 27 participants representing 13 services elaborated the "Brazilian Guidelines for the Diagnosis and Treatment of Hereditary Angioedema - 2017" 3,4. In the 2017 guidelines, the flowcharts and algorithms for the diagnosis and treatment of HAE were updated, with the inclusion of new drugs that became available in Brazil. The aim of the document is to standardize the management of HAE in the country, addressing the causes, classification, clinical features, warning signals, diagnostic criteria, diagnostic laboratory tests, differential diagnosis, long- and short-term prophylaxis, and acute attack therapy. We would like to highlight some aspects of the 2017 guidelines:

1. HAE is underdiagnosed and may have a higher prevalence than the currently estimated rate in the literature, especially if we consider the HAE subtype with normal C1-INH.
2. The document is innovative when it proposes warning signals for HAE awareness, with the acronym "HAAAAE" that facilitates disease recognition by health professionals. HAAAAE stands for Heredity, recurrent Angioedema, recurrent Abdominal pain, Absence of urticaria, Absence of response to antihistamines and association with Estrogen.
3. Always giving priority to clinical history, the measurement of C4 levels is the main screening test for HAE with C1 inhibitor deficiency.
4. Angioedema may be a clinical manifestation of anaphylaxis or constitute a clinical entity by itself, and, in this case, it may be considered a syndrome. The present guidelines describe a classification of angioedema based on two main endotypes: the angioedema due to excess bradykinin and the histaminergic angioedema, associated with degranulation of basophils/mast cells.
5. Four variables should be analysed when considering long-term prophylaxis: the frequency, severity, and impact of HAE attacks on patients’ quality of life, as well as the availability of proper medications to treat HAE attacks, which may occur even with the prescription of long-term prophylaxis. The development of new drugs to treat HAE attacks was a major advance. Most likely, the next breakthrough development will be new drugs for HAE long-term prophylaxis.
6. The drug of choice for short-term prophylaxis, when indicated, is plasma-derived C1-INH concentrate (pdC1-INH).
7. There was great progress with Icatibant and pdC1-INH concentrate approval in Brazil, which are the drugs of choice for HAE attack treatment. These therapies can be used at home and should be available for all patients.

The authors of the "Brazilian Guidelines for the Diagnosis and Treatment of Hereditary Angioedema - 2017" hope that all readers enjoy the guidelines, which have been divided in two parts; part one is being published in the present issue, and part two will be published in a future issue. The guidelines were developed to assist with the diagnosis and treatment of HAE, to reduce HAE morbidity and mortality and to improve patients’ quality of life.
